# HPV genotype distribution according to severity of cervical neoplasia using the digene HPV genotyping LQ test

**DOI:** 10.1007/s00705-012-1584-4

**Published:** 2013-01-09

**Authors:** Philippe Halfon, Maria Luisa Mateos Lindemann, Audrey Raimondo, Sophie Ravet, Claire Camus, Hacène Khiri, Guillaume Pénaranda, Mario Sideri, Maria Teresa Sandri

**Affiliations:** 1 Laboratoire Alphabio, 23 Rue de Friedland, 13006 Marseille, France; 2 Unité de Virologie, Hôpital Ambroise Paré, Marseille, France; 3 Servicio de Microbiología, Hospital Ramón y Cajal, Madrid, Spain; 4 Laboratory Medicine Unit, European Institute of Oncology, Milan, Italy

## Abstract

A new genotyping-based DNA assay (Digene LQ^®^) was developed recently. The primary aim was to assess the distribution of HPV types using this new assay in atypical squamous cells of undeterminate significance (ASCUS). The secondary aim was to correlate the HPV types with the severity of the disease. The study population comprised 376 ASCUS women. The women were all Hybrid Capture II (HCII) positive and were admitted in three European referral gynecology clinics between 2007 and 2010. A colposcopy with histological examination was performed in all these patients. HPV 16 was typed in 40 % of patients, HPV 18 in 7 %, and HPV 31 in 17 %, and 18 % of patients had mixed genotypes. Patients aged over 30 more often had the HPV 16 genotype than patients aged under 30 (29 % vs. 11 %, chi-square test p < 0.001). The risk of cervical intra-epithelial neoplasia of grade 2 or more (CIN2 +) when HPV 18 positive is lower than the probability associated with HPV 16 or HPV 31: 28 % vs. 58 % and 52 %, respectively (chi-square test, p = 0.005 and p = 0.05, respectively). The Digene LQ^®^, a new sequence-specific hybrid capture sample preparation, is fast and efficient and allows high-throughput genotyping of 18 HR HPV types by PCR compared to traditional non-sequence-specific sample preparation methods.

## Introduction

High-risk types of human papillomavirus (HPV) are causative agents for cervical cancer. The effectiveness of the cervical screening program could be improved by testing for the DNA of high-risk types of HPV as a primary screening tool [1–3]. More than 200 genotypes have been identified, among which about 40 can infect the mucosa of the anogenital tract. HPV genotypes are classified into “high-risk” HPV (HR HPV), “probable high-risk” HPV, and “low-risk” HPV (LR-HPV) genotypes [3–5]. The classification has been updated, based on epidemiological data. The HR group includes 15 HPV genotypes (types 16, 18, 31, 33, 35, 39, 45, 51, 52, 56, 58, 59, 68, 73, 82) that proved to be associated with cervical cancer, while the LR group includes 12 HPV genotypes (types 6, 11, 40, 42, 43, 44, 54, 61, 70, 72, 81, and CP6108) that are not potentially oncogenic and not involved in the development of cervical cancer [6, 7]. In a recent study, Bouvard et al. [8] suggested that the following 12 genotypes might be HR types: types 16, 18, 31, 33, 35, 39, 45, 51, 52, 56, 58, and 59. Three HPV genotypes (types 26, 53, 66) are classified as probable HR genotypes because their association with cervical cancer is very difficult to assess, giving a low number of related cases [9]. Some authors have proposed that HPV genotypes 26, 53, 66, 73, and 82 should be added to the HR genotypes classification associated with cervical cancer [5, 10].

In view of the increasing importance of HPV genotyping, it is important to develop robust, high-throughput assays. Recently, HPV genotyping tests have been presented as relevant for management of screened women, in order to identify which HPV-positive women have persistent oncogenic HPV infection [10, 11]. Studies have shown that a single positive test result from either type 16 or type 18 has high predictive value for cervical intra-epithelial neoplasia grade 2 or more (CIN2+) [4, 5, 12]. US guidelines recommend genotyping for type 16 and type 18 in HR HPV-positive women over 30 years of age with normal PAP to determine whether immediate colposcopy is needed [13]. Several genotyping assays have been developed based on different technologies (reverse dot blot, biotinylated MY09/11, DNA-chip technology). These commercial assays allow the detection of 37 HPV genotypes (LA, Roche), 24 HPV genotypes (Papillocheck HPV-screening test, Greiner Bio-One), 35 HPV genotypes (Clinical Arrays, CLART Genomica), and 28 HPV genotypes (INNO-LiPA HPV Genotyping, Innogenetics) [14–17]. The most used genotyping assay, LA, correlates in performance with the HPV Hybrid Capture II (HCII) test for detection of CIN 2+ [18–20].

A novel, commercial system (Digene LQ^®^) for the identification of 18 HR HPV types on GP5+/6+-PCR products was developed and compared analytically to the established Reverse Line Blot (RLB) genotyping assay [21]. Godinez et al. [22] recently performed clinical validation of QIAGEN LQ in women > 40 years old. However, no clinical validation has been performed in women aged > 18 years old.

The primary aim of this study was to assess the distribution of HPV types using this new assay. The secondary aim was to correlate the HPV types to the severity of the disease (cytology and histology) with calculation of probabilities of CIN2+ by HPV type.

## Materials and methods

### Patients

The study population comprised 376 atypical squamous cells of undetermined significance (ASCUS) women who were admitted to three European referral gynecology clinics between 2007 and 2010: 158 patients from Madrid (Spain), 123 from Marseille (France), and 95 from Milan (Italy). Criteria for eligibility were age between 18 and 60 years, abnormal cervical smears and HC-II-positive results, and referral for colposcopy with histology. Before colposcopy, a cervical sample was obtained using the ThinPrep method (Cytyc France Sarl, Roissy, France). The cervical scrapes were collected with the PreservCyt transport medium (Cytyc Corp., Marlborough, MA). All of these tests were performed on the samples collected in PreservCyt liquid medium for liquid-based cytology (ThinPrep). Informed consent was obtained from each participant according to the ethics committee guidelines. This study was approved by the CPP Sud-Med I (Comité de Protection des Personnes Sud Méditerranée I) under reference number 07 22.

The following assays were carried out and scored in strict accordance with the manufacturer’s instructions.

### Hybrid capture II

(HCII) (Digene): this assay detects 13 HR HPV genotypes (HPV16, 18, 31, 33, 35, 39, 45, 51, 52, 56, 58, 59, 68). The HCII test was performed using the automated HCII assay system as described previously (13). For each specimen, results were expressed in relative light unit/cutoff (RLU/CO), corresponding to the ratio of the specimen luminescence relative to the luminescence of the 1.0 pg/ml HPV16 standard provided with the kit. Samples with an RLU/CO value ≥1 were considered HCII-HR positive. Samples with an RLU/CO value <1.0 were considered HCII-HR negative.

### Digene HPV genotyping LQ Test (Digene LQ test) analysis

The Digene LQ test utilizes probes for 18 HR HPV types (i.e., HPV 16,18, 26, 31, 33, 35, 39, 45, 51, 52, 53, 56, 58, 59, 66, 68, 73, 82) that are the same as the respective RLB probes, with minor modifications, and are immobilized on color-coded beads. The Digene LQ test detection was performed in the Luminex 100 IS System (Luminex Corporation). In brief, 3B buffer was added to the HR HPV beads to minimize the background in the final Luminex read-out. Subsequently, GP5+/6+-PCR products were added. Next, heat-denaturation, hybridization under stringent conditions, and incubation with streptavidin-conjugated R-phycoerythrin detection conjugate were followed by read-out according to the specified instrument settings, resulting in MFI levels per HPV type for each specimen.

### Statistical analysis

Two-sided P-values were calculated by Chi-square or Fisher exact tests and placed on 2 × 2 contingency tables; Cochran-Armitage test for trend was used for testing trend binomial age proportions across levels of cervical intra-epithelial neoplasia. All P-values < 0.05 were considered statistically significant. Calculations were performed using SAS software (SAS Institute Inc., Cary, NC).

## Results

Table 1 shows the key characteristics of the 376 ASCUS women. The mean age was 37 (± 11) years (116 were <30 years old, and 260 were ≥30 years old); 167 (44 %) were CIN2+, and 67 (18 %) were CIN3+. Digene LQ was found positive in 349 (93 %) patients and negative in 27 (7 %).

**Table 1:** Key characteristics of the 376 ASCUS+ patients

Characteristics	All ASCUS + patients (N = 376)	Madrid – Spain (N = 158)	Marseille – France (N = 123)	Milan – Italy (N = 95)
Age; mean (± Sd)	37 (± 11)	36 (± 11)	37 (± 12)	39 (± 10)
Smear – N (%)				
ASCUS	55 (15)	8 (5)	44 (36)	2 (2)
LSIL	169 (45)	87 (55)	48 (39)	35 (37)
HSIL	151 (40)	63 (40)	30 (24)	58 (61)
Cancer	1 (-)	0 (-)	1 (-)	0 (-)
Biopsy – N (%)				
Normal	48 (13)	11 (7)	24 (20)	13 (14)
CIN1	161 (43)	79 (50)	54 (44)	28 (29)
CIN2	100 (27)	41 (26)	33 (27)	27 (28)
CIN3	65 (17)	27 (17)	11 (9)	27 (28)
Cancer	2 (-)	0 (-)	1 (-)	0 (-)
Qiagen HPV LQ – N (%)				
Positive (all types)	349 (93)	146 (92)	118 (96)	85 (89)
Negative	27 (7)	12 (8)	5 (4)	10 (11)
HCII – N (%)				
Positive (all types)	376 (100)	158 (100)	123 (100)	95 (100)

**Fig. 1:**
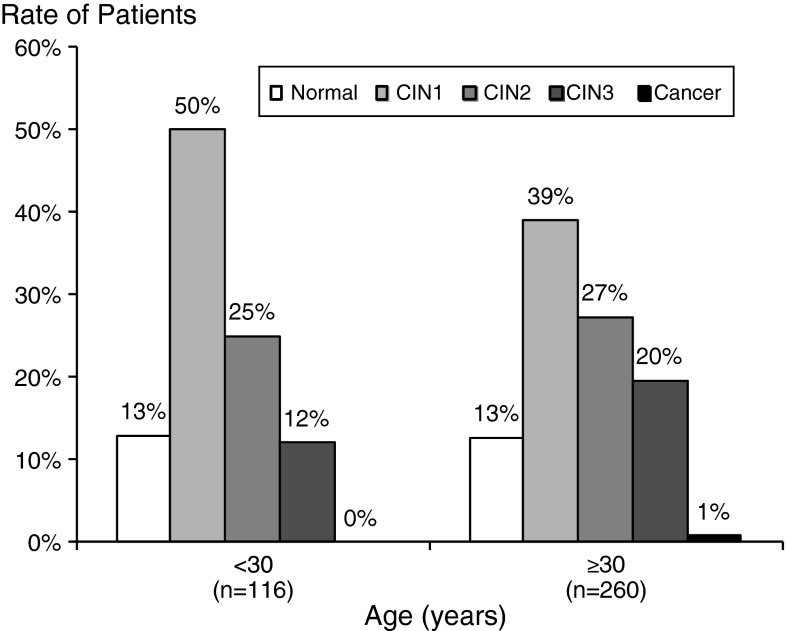
Histologic distribution among patients in the < 30- vs. ≥30-year-old age groups. Bars show the rate of patients with “normal” histology (white bars), “CIN1″ histology (light grey bars), or CIN 2, CIN3, or cancer (black bars)) among patients under 30 years old on one side, and over 30 years old on the other side. There was no significant correlation between the rate of histologic disease and age group (Cochran-Armitage test for trend p = 0.20)

Figure 1 shows the histologic distribution among patients in the age group <30 vs. those ≥30 years old. There was no significant correlation between the rate of histologic disease and age group: 25 % (29/116) of <30-year-old patients were CIN2,^1^ compared with 27 % (71/260) of ≥30-year-old patients. Similarly, 12 % (14/116) of <30-year-old patients were CIN3 compared with 20 % (51/260) of ≥30-year-old patients (p-value not significant). The QIAGEN LQ test gave a positive result for 349 patients, and thus, the concordance between Digene LQ and HCII was 93 %.

**Fig. 2:**
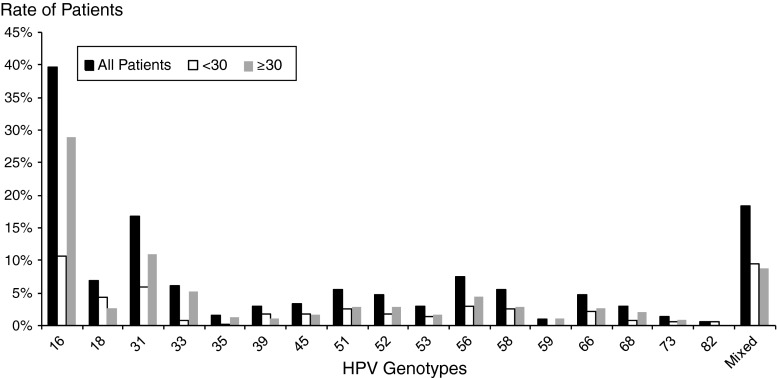
Distribution of HPV genotypes using LQ. Black bars, all 376 patients; white bars, patients aged under 30; grey bars, patients aged over 30. Patients aged over 30 more often had HPV 16 genotype than patients aged under 30 (29 % vs. 11 %, chi-square test p < 0.001)

Figure 2 shows the distribution of HPV genotypes using Digene LQ in all patients, in patients aged under 30 years old and in patients aged over 30 years old. Among HPV genotypes, HPV16 was typed in 40 % of patients, HPV 18 in 7 %, HPV31 in 17 %, and HPV 56 in 7 %, and 18 % of patients had mixed genotypes. Among the infections with multiple HPV genotypes (N = 69), 52 (76 %) had two HPV genotypes, 16 (23 %) had three HPV genotypes, and 1 (1 %) had five HPV genotypes. Patients aged over 30 more often had the HPV16 genotype than patients aged under 30 (29 % vs. 11 %, Chi square test p < 0.001).

**Fig. 3:**
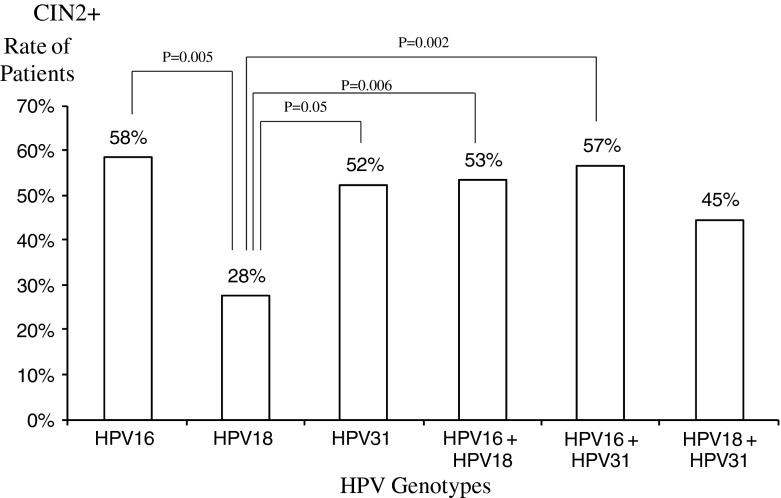
Risk of CIN2+ according to HPV type. Bars show the rate of patients with the corresponding HPV type among patients with CIN2+ histology. Groups were compared with each other; chi-square test p-values are shown on the line that links the *two bars*

**Fig. 4:**
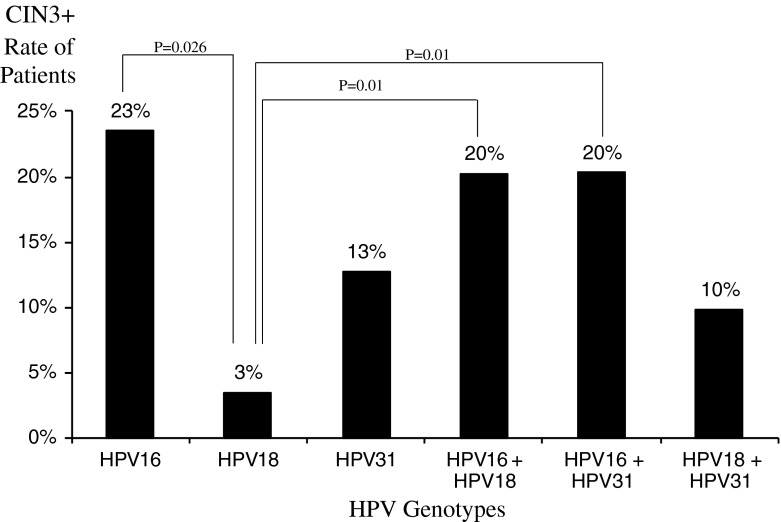
Risk of CIN3+ according to HPV type. Bars show the rate of patients with the corresponding HPV type among patients with CIN3+ histology. Groups were compared with each other; chi-square test p-values are shown on the line that links the two bars

Figure 3 shows the risk of CIN2+ according to HPV type. The risk of CIN2+ when HPV 18 is positive is lower than the probability associated with HPV16 or HPV31: 28 % vs. 58 % and 52 %, respectively (Chi square test, p = 0.005 and p = 0.05, respectively). Similarly, the probability of CIN3+ when HPV18 is positive is lower than probability associated with HPV16 but not with HPV31: 3 %, 23 %, and 13 %, respectively (Chi square test, p = 0.026 and p = 0.26, respectively) (Fig. 4).

**Fig. 5:**
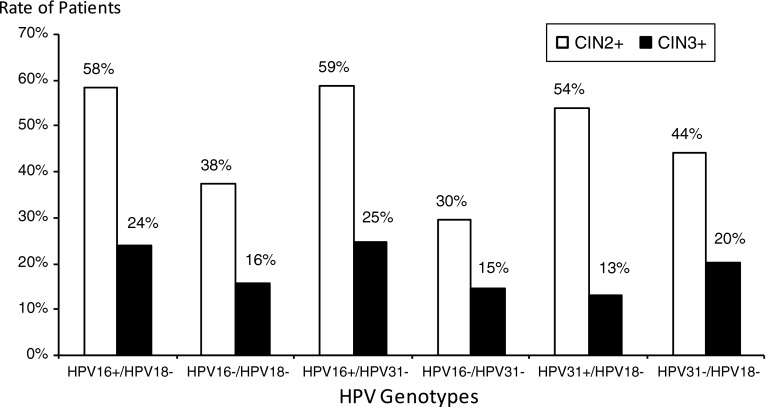
Risk of CIN2+ and CIN3+ according to HPV type. Bars show the rate of patients with the presence or absence of corresponding HPV types: “+” stands for presence of the HPV type, “−”stands for absence of the HPV type (HPV16+/HPV18- stands for presence of HPV 16 and absence of HPV 18). White bars are CIN2+ patients, and black bars are CIN3+ patients

Figure 5 shows the risk of CIN2+ or CIN3+ according to the combination of HPV genotypes. The risk of CIN2+ when neither HPV16 nor HPV31 is detected is lower than in all other cases (30 %; Chi square, p < 0.05). Similarly, the risk of CIN2 + when neither HPV16 nor HPV18 is detected is lower than in other cases except HPV18 negative and HPV31 negative (38 % vs. 44 % respectively, Chi square p = 0.10). The risk of CIN3+ when HPV16 is detected, but not HPV18, and the risk of CIN3+ when HPV16 is detected, but not HPV31, are higher than in all other cases, except when neither HPV18 nor HPV31 is detected (24 % and 25 % compared with 20 %, respectively).

Table 2 shows the age distribution of HPV genotypes among CIN1 and CIN2+ patients. A significant difference in age distribution was observed in patients with high-risk HPVs (by pooling HPV16, HPV18, HPV31, and other HPV types) (p = 0.04): an increasing rate of CIN2+ was observed with increasing age (inversely for CIN 1 patients). Similarly, a significant difference in age was observed in patients with “other” HPV types (other than HPV16, HPV18, or HPV31) (p = 0.04).

**Table 2:** HPV genotype distribution according to age CIN1 or CIN2+

	Age (years)	CIN1	CIN2+	P
HPV 16/18/31 Positive	<20	2 (67 %)	1 (33 %)	0.17
	20-29	25 (49 %)	26 (51 %)	
	30-39	29 (40 %)	43 (60 %)	
	40-49	16 (38 %)	26 (62 %)	
	≥50	5 (36 %)	9 (64 %)	
Other HPV	<20	1 (100 %)	0 (-)	0.04
	20-29	24 (71 %)	10 (29 %)	
	30-39	19 (73 %)	7 (27 %)	
	40-49	12 (46 %)	14 (54 %)	
	≥50	8 (53 %)	7 (47 %)	
Overall HPV Positive	<20	3 (75 %)	1 (25 %)	0.04
	20-29	49 (58 %)	36 (42 %)	
	30-39	48 (49 %)	50 (51 %	
	40-49	28 (41 %)	40 (59 %)	
	≥50	13 (45 %)	16 (55 %)	

## Discussion

This multicenter study evaluated for the first time the clinical utility of HPV genotyping using the recently developed Digene LQ^®^ assay (in atypical squamous cells of undetermined significance, and HCII-positive women referred for colposcopy in three European centers [France, Spain, and Italy]). The distribution of genotypes using this assay indicated, as expected, that HPV types 16, 31/33, 18 and mixed genotypes are the most prevalent, in accordance with previously reported results [23].

The recent ASCCP guidelines recommend the use of HPV genotyping for patient management, with a direct referral to colposcopy based on HPV16- and/or HPV18-positive results [13]. Moreover, screening with HPV DNA testing for oncogenic genotypes followed by cytological triage has attractive features that may serve the screening needs for a post-vaccination era in the US. However, the methods for HPV typing should be specifically validated with CIN2+ as a clinical endpoint, and comparatively with other methods. This would be a prerequisite for the use of genotyping assays in cervical cancer screening algorithms. Particularly in light of the recent FDA approval of an HPV genotyping test, this study focused on how typing could be used to assist clinical decisions and whether its implementation would be cost-effective [24, 25].

This new sequence-specific Digene LQ test is fast and efficient and allows direct HPV genotyping of 18 HR types by PCR, compared to traditional non-sequence-specific test methods. The utility of this method was evaluated on cervical samples positive for HR HPV by the HCII screening assay from patients referred for colposcopy.

Among women with ASCUS cytology, the relative probability of pre-cancer is higher in HR HPV-positive women than HR HPV-negative women [20]. Interestingly, using the Digene LQ^®^, the probability of being CIN2+ when having HPV16 is 58 %, similar to the probability associated with HPV31, 52 %, and the probability is 28 % if HPV18 positive. The probability of being CIN3+ when having HPV16 is 23 % (the probability is 3 % if HPV18 positive, and 13 % if HPV31 positive). The relative probability of CIN2+ when being HPV16+/HPV18- is 58 % (the probability of CIN3+ is 24 %). Similarly, the probability of CIN2+ when being HPV16+/HPV31 is 59 % (the probability of CIN3+ is 25 %), and the probability of CIN2+ when being HPV31+/HPV18- is 54 % (the probability of CIN3+ is 13 %).

These data are similar to those reported in the recently published ATHENA study: HPV16/HPV18+ women had a greater probability of CIN 2 or worse compared with pooled HR-HPV+ and HR-HPV- women (24.4 %, 14.0 %, and 0.8 %, respectively) [20].

Among HR-HPV-positive women, risk of disease likely increases over time, as has been reported for genotypes 16 and 18 by Khan et al. [12]. Up to now, the risk threshold for performing colposcopy in response to specific cytology and HPV test results has not been defined. Castle et al. suggested that women with a probability for CIN3+ disease of 10 % or greater over two years should be investigated [26].

While all women with ASCUS cytology testing positive for HR HPV should be referred for colposcopy, the results from this study demonstrate that those women with ASCUS cytology testing positive with HPV16 and/or 18 and 31 are at a particularly high risk for ≥ CIN3 disease, reinforcing the need for immediate colposcopy and more intense follow-up, particularly in the case of a negative colposcopy. These findings corroborate those of other studies [27, 28].

This study points out the greater probability of CIN2 and CIN3 for patients testing positive for HPV31. HPV31 is a more prevalent type than HPV18 in the ASCUS population in European countries [19, 29]. Notably, this study highlights that the probability of HPV31 is greater than that of HPV18. This was also observed by Söderlund et al. [30]: an odds ratio of the risk of CIN2+ or CIN3+ was 3.79 and 2.83 for HPV31 vs. 1.46 and 0.82 for HPV18, respectively.

Another prevalent genotype in our population was HPV56, found in the same proportion as HPV18.

Regarding the laboratory technical characteristics, this assay uses multiplex, bead-based xMAP technology and an automated, high-throughput read-out by either the LiquiChip 200 workstation (QIAGEN, Hilden, Germany) or Luminex 100 IS System (Luminex Corporation, Austin, TX). The test was developed for identification of the 18 HR HPV genotypes associated with cervical cancer using GP5+/6+-PCR products. The Digene LQ Test and the RLB assay have been reported to have a high level of agreement in detection and genotyping of 18 HR HPV types in HCII-positive specimens [21].

## Conclusions

The Digene LQ^®^, a new sequence-specific HC sample preparation is fast and efficient and allows high-throughput genotyping of 18 HR HPV types by PCR compared to traditional non-sequence-specific sample preparation methods. This study showed that HPV types 16, 31, 33, 18 and mixed genotypes are the most prevalent genotypes and that ASCUS women who are HPV16 positive have the highest probability of CIN2+ and CIN3+.

## Footnotes

1 Cervical intraepithelial neoplasia (CIN) is graded according to its pathologic progress, from CIN 1 to CIN 3. CIN 2 + stands for CIN 2 and CIN 3 grades.
